# Supplementary material for: Genetic and chemotherapeutic influences on germline hypermutation
Source: Nature. 2022 May 11;605(7910):503–8. doi: 10.1038/s41586-022-04712-2 (PMC9117138; doi:10.1038/s41586-022-04712-2)
Supplement: Supplementary file 1 — Supplementary Figs. 1–7, Supplementary Tables 2 and 5–9, descriptions of Supplementary Tables 1, 3 and 4, Supplementary References and Supplementary Notes, detailing the GEL consortia members. [file 41586_2022_4712_MOESM1_ESM.pdf]

nature portfolio

https://doi.org/10.1038/s41586-022-04712-2

Supplementary information

Genetic and chemotherapeutic influences on germline hypermutation

In the format provided by the authors and unedited

Supplementary Note

The Genomics England Research Consortium

Ambrose, J. C.[1] ; Arumugam, P.[1] ; Bevers, R.[1] ; Bleda, M.[1] ; Boardman-Pretty, F.[1,2] ; Boustred, C. R.[1] ; Brittain, H.[1] ; Caulfield, M. J.[1,2] ; Chan, G. C.[1] ; Fowler, T.[1] ; Giess A.[1]; Hamblin, A.[1] ; Henderson, S.[1,2]; Hubbard, T. J. P.[1] ; Jackson, R.[1] ; Jones, L. J.[1,2]; Kasperaviciute, D.[1,2] ; Kayikci, M.[1] ; Kousathanas, A.[1]; Lahnstein, L.[1] ; Leigh, S. E. A.[1] ; Leong, I. U. S.[1] ; Lopez, F. J.[1] ; Maleady-Crowe, F.[1] ; McEntagart, M.[1]; Minneci F.[1] ; Moutsianas, L.[1,2] ; Mueller, M.[1,2] ; Murugaesu, N.[1]; Need, A. C.[1,2] ; O'Donovan P.[1]; Odhams, C. A.[1] ; Patch, C.[1,2] ; Perez-Gil, D.[1] ; Pereira, M. B.[1] ; Pullinger, J.[1] ; Rahim, T.[1] ; Rendon, A.[1] ; Rogers, T.[1] ; Savage, K.[1] ; Sawant, K.[1]; Scott, R. H.[1]; Siddiq, A.[1] ; Sieghart, A.[1] ; Smith, S. C.[1]; Sosinsky, A.[1,2] ; Stuckey, A.[1] ; Tanguy M.[1] ; Taylor Tavares, A. L.[1]; Thomas, E. R. A.[1,2] ; Thompson, S. R.[1] ; Tucci, A.[1,2] ; Welland, M. J.[1] ; Williams, E.[1] ; Witkowska, K.[1,2] ; Wood, S. M.[1,2].

1. Genomics England, London, UK
2. William Harvey Research Institute, Queen Mary University of London, London, EC1M 6BQ, UK.

Supplemental Figures

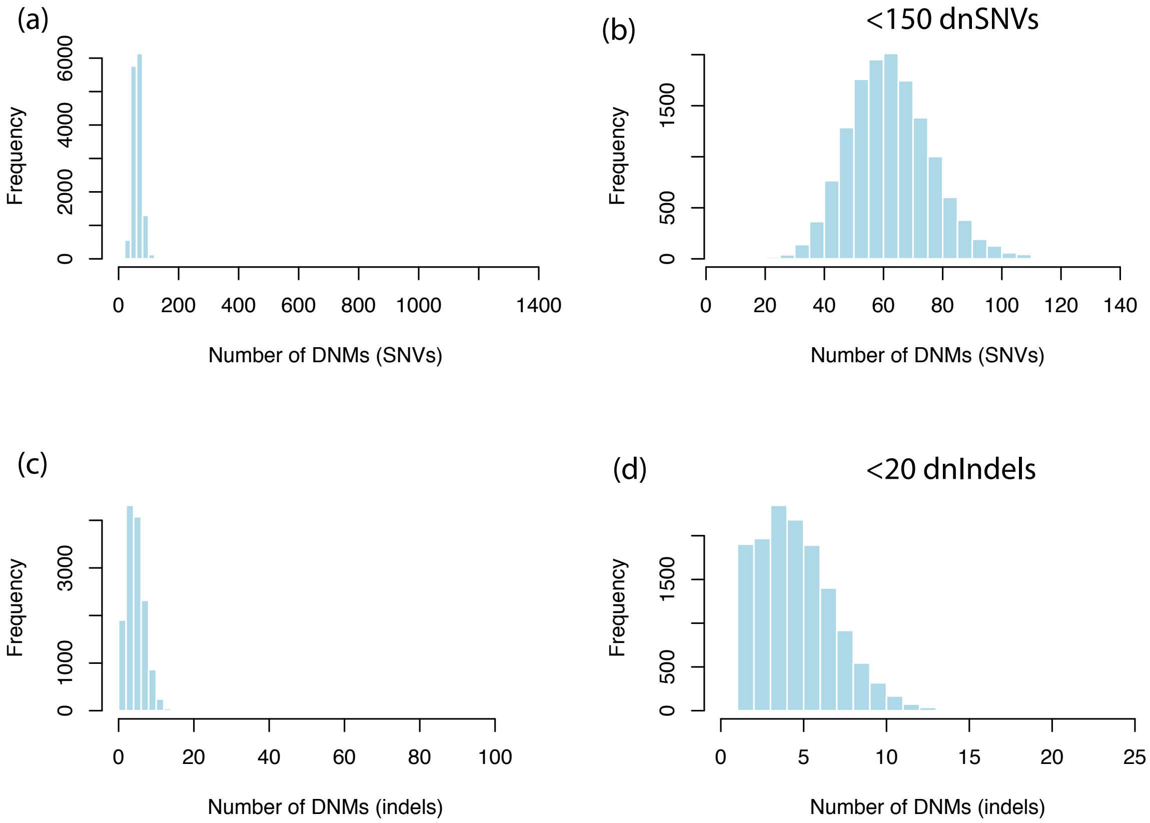

Supplemental Figure 1: Distribution of number of *de novo* SNVs for all individuals (a) and those with <150 DNMs (b). Distribution of number of *de novo* InDels per person for all individuals (c) and those with <20 indels (d).

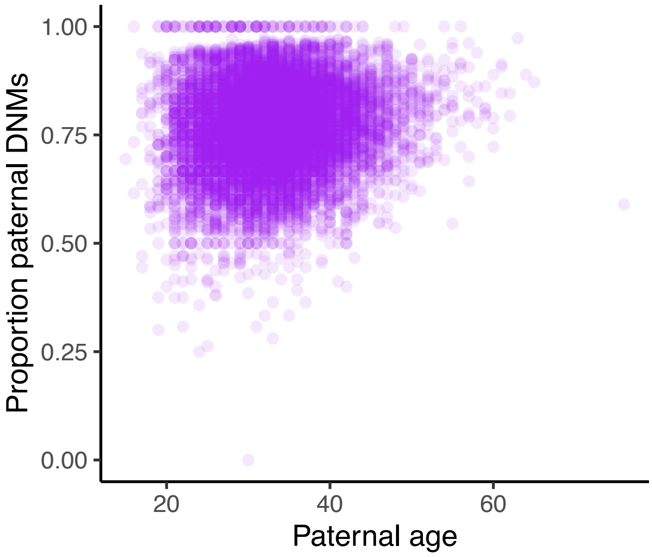

Supplemental Figure 2: Proportion of paternally phased DNMs against paternal age. X-axis refers to paternal age at child's birth. Y-axis is the proportion of phased DNMs that phased paternally.

(a)

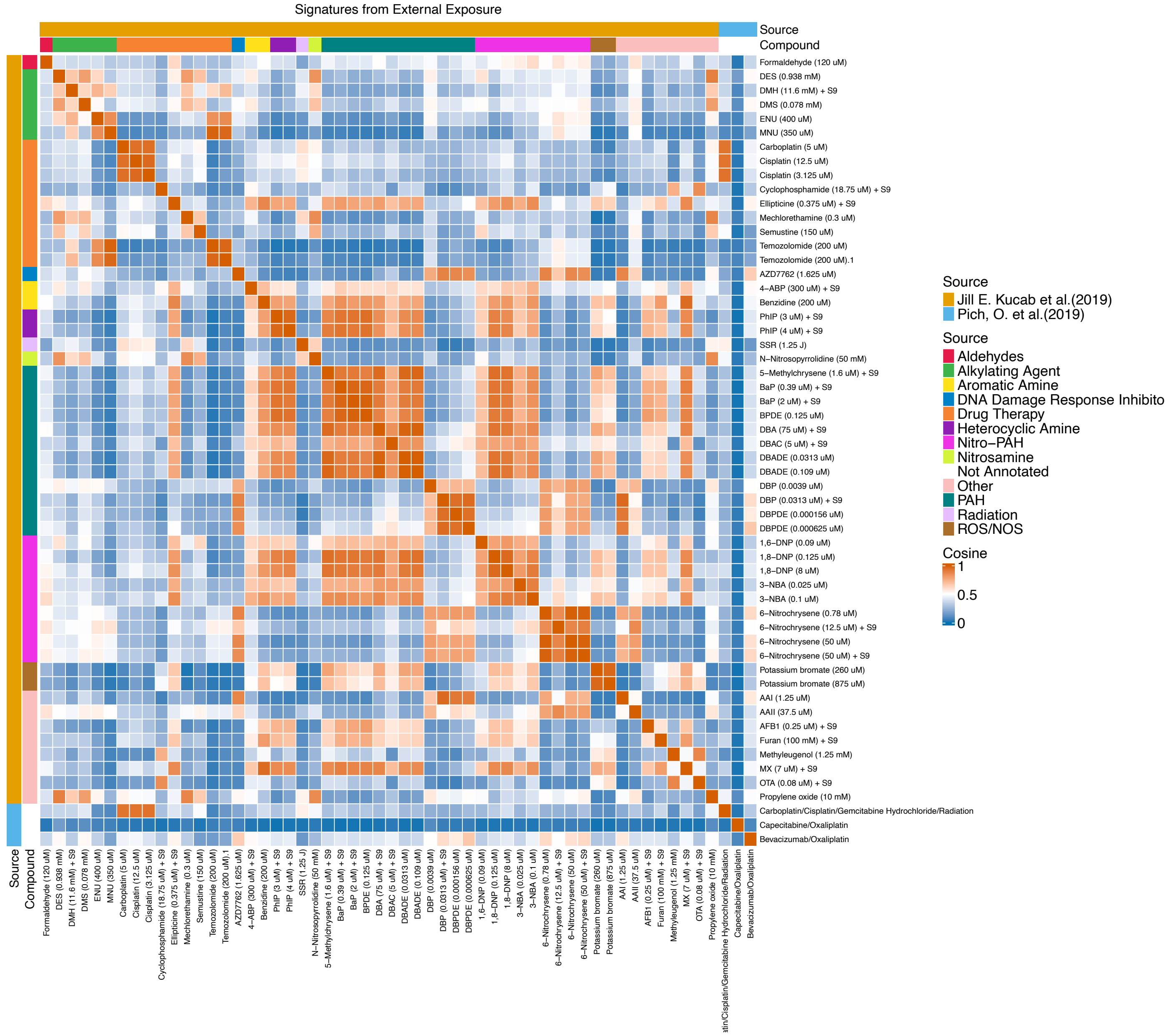

Signatures from External Exposure
Source
Compound
Formaldehyde (120 uM)
DES (0.938 mM)
DMH (11.6 mM) + S9
DMS (0.078 mM)
ENU (400 uM)
MNU (350 uM)
Carboplatin (5 uM)
Cisplatin (12.5 uM)
Cisplatin (3.125 uM)
Cyclophosphamide (18.75 uM) + S9
Ellipticine (0.375 uM) + S9
Mechlorethamine (0.3 uM)
Semustine (150 uM)
Temozolomide (200 uM)
Temozolomide (200 uM).1
AZD7762 (1.625 uM)
4–ABP (300 uM) + S9
Benzidine (200 uM)
PhIP (3 uM) + S9
PhIP (4 uM) + S9
SSR (1.25 J)
N–Nitrosopyrrolidine (50 mM)
5–Methylchrysene (1.6 uM) + S9
BaP (0.39 uM) + S9
BaP (2 uM) + S9
BPDE (0.125 uM)
DBA (75 uM) + S9
DBAC (5 uM) + S9
DBADE (0.0313 uM)
DBADE (0.109 uM)
DBP (0.0039 uM)
DBP (0.0313 uM) + S9
DBPDE (0.000156 uM)
DBPDE (0.000625 uM)
1,6–DNP (0.09 uM)
1,8–DNP (0.125 uM)
1,8–DNP (8 uM)
3–NBA (0.025 uM)
3–NBA (0.1 uM)
6–Nitrochrysene (0.78 uM)
6–Nitrochrysene (12.5 uM) + S9
6–Nitrochrysene (50 uM)
6–Nitrochrysene (50 uM) + S9
Potassium bromate (260 uM)
Potassium bromate (875 uM)
AAI (1.25 uM)
AAII (37.5 uM)
AFB1 (0.25 uM) + S9
Furan (100 mM) + S9
Methyleugenol (1.25 mM)
MX (7 uM) + S9
OTA (0.08 uM) + S9
Propylene oxide (10 mM)
Carboplatin/Cisplatin/Gemcitabine Hydrochloride/Radiation
Capecitabine/Oxaliplatin
Bevacizumab/Oxaliplatin
Source
Jill E. Kucab et al.(2019)
Pich, O. et al.(2019)
Source
Aldehydes
Alkylating Agent
Aromatic Amine
DNA Damage Response Inhibito
Drug Therapy
Heterocyclic Amine
Nitro–PAH
Nitrosamine
Not Annotated
Other
PAH
Radiation
ROS/NOS
Cosine
1
0.5
0

(b)

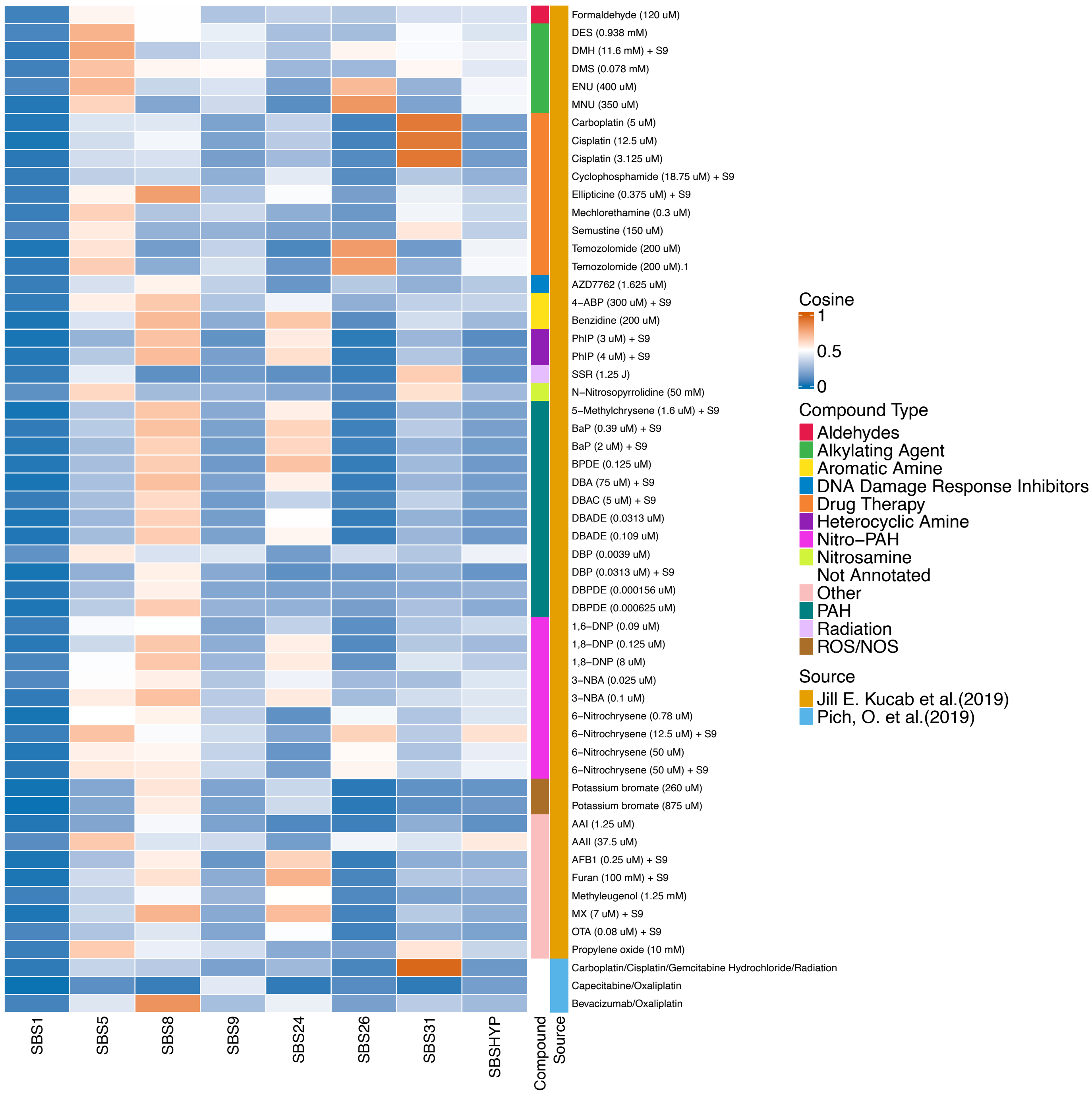

Supplemental Figure 3:

(a) Cosine similarity of all the signatures caused by environmental mutagens amongst themselves.

(b) Cosine similarity of all the signatures caused by environmental mutagens with the extracted signatures from the hypermutated individuals. These signatures were compiled from Kucab et al 2019, Pich et al 2019 and Volkova et al 2020 (see Methods)

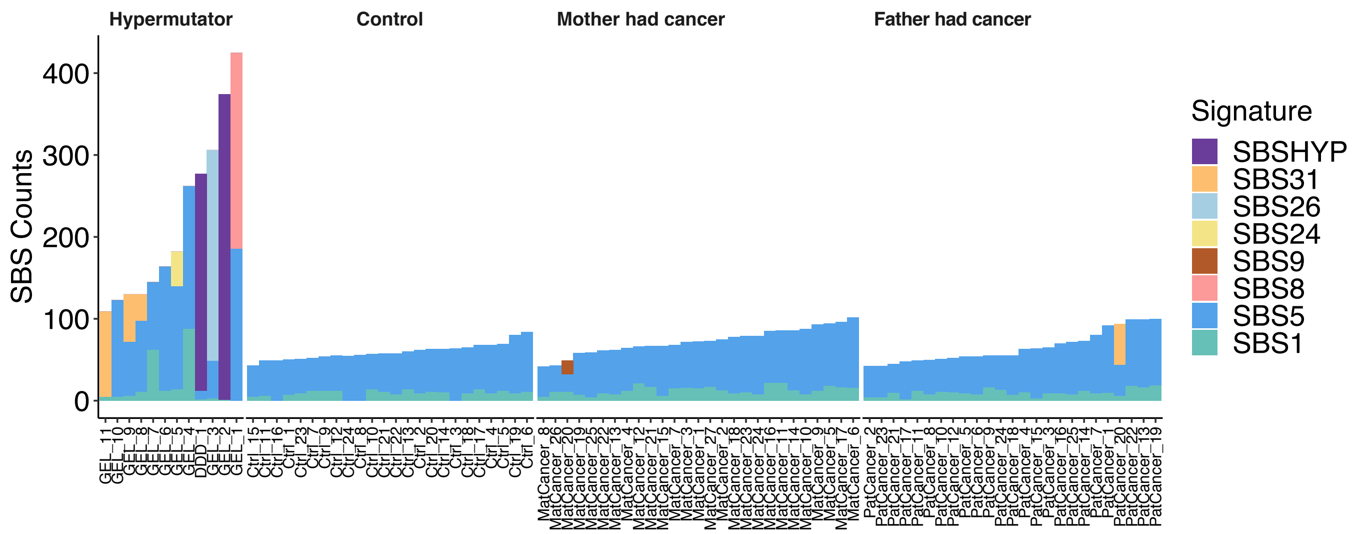

Supplemental Figure 4: Mutational signature contributions for hypermutators, a set of controls selected matched on parental age and individuals who have a parental history of cancer.

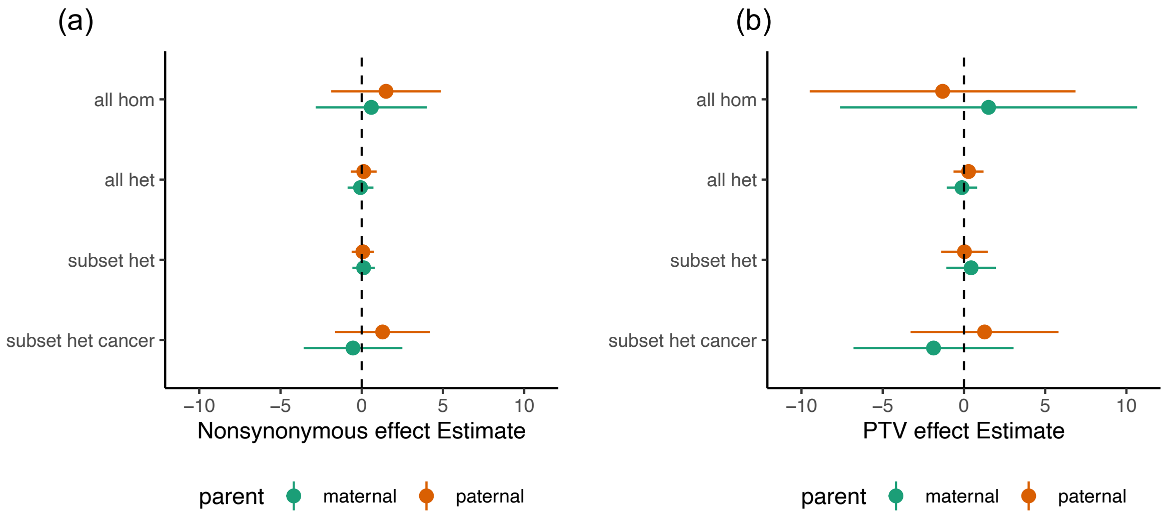

Supplemental Figure 5: Impact of rare variants in DNA repair genes on germline mutation rate. Poisson regression effect estimates for binary variables of having a parental variant in genes known to be involved in DNA repair. (a) considered all nonsynonymous variants in the subsets (b) is restricted to PTVs.

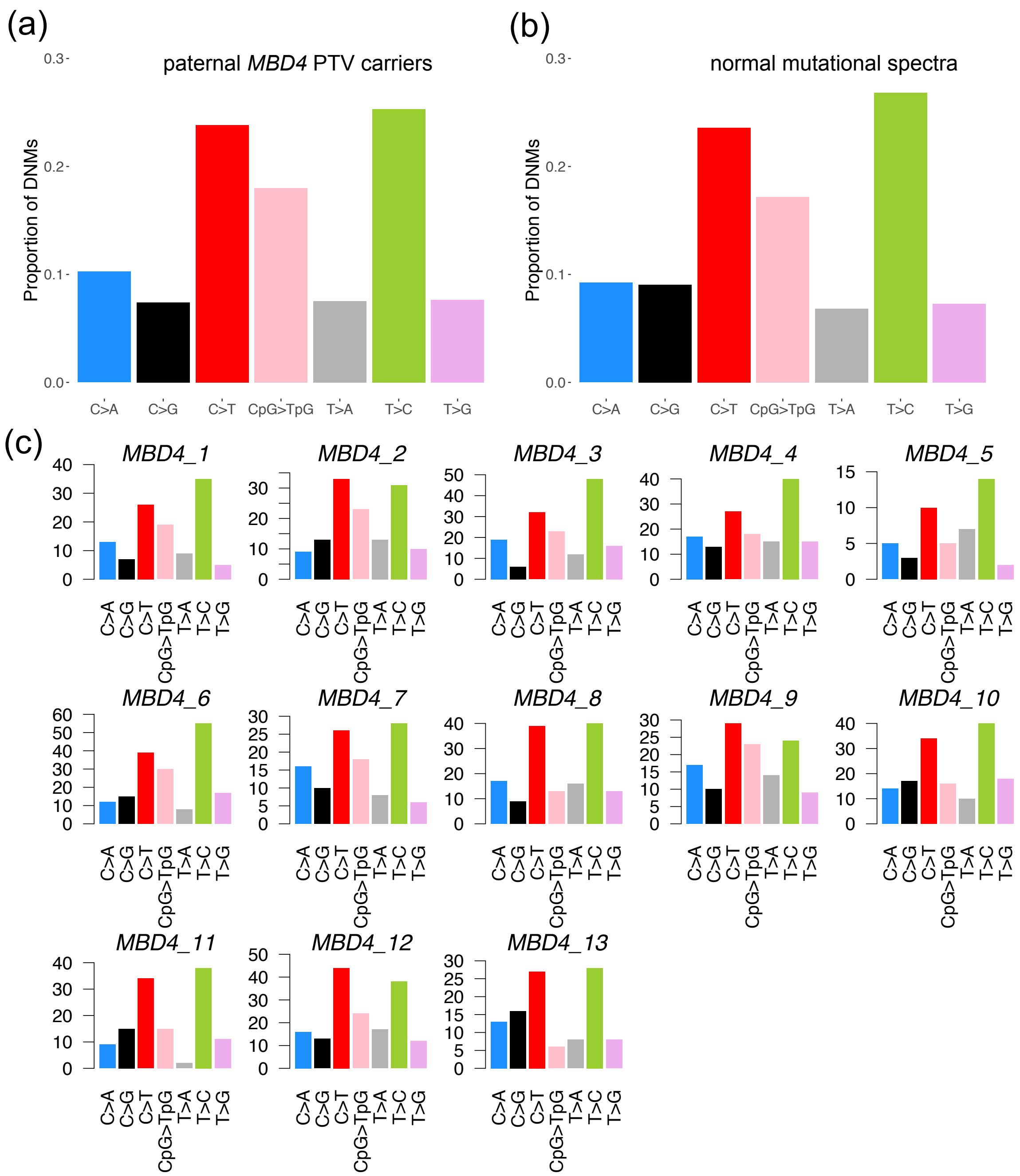

Supplemental Figure 6: Comparing the mutational spectra of DNMs across the 13 paternal *MBD4* paternal PTV carriers (a) with the expected proportion of mutations (b) in each mutation type taken from Rahbari et al. (c) The individual mutational spectra demonstrating that no one individual has an elevated number of CpG>TpG mutations.

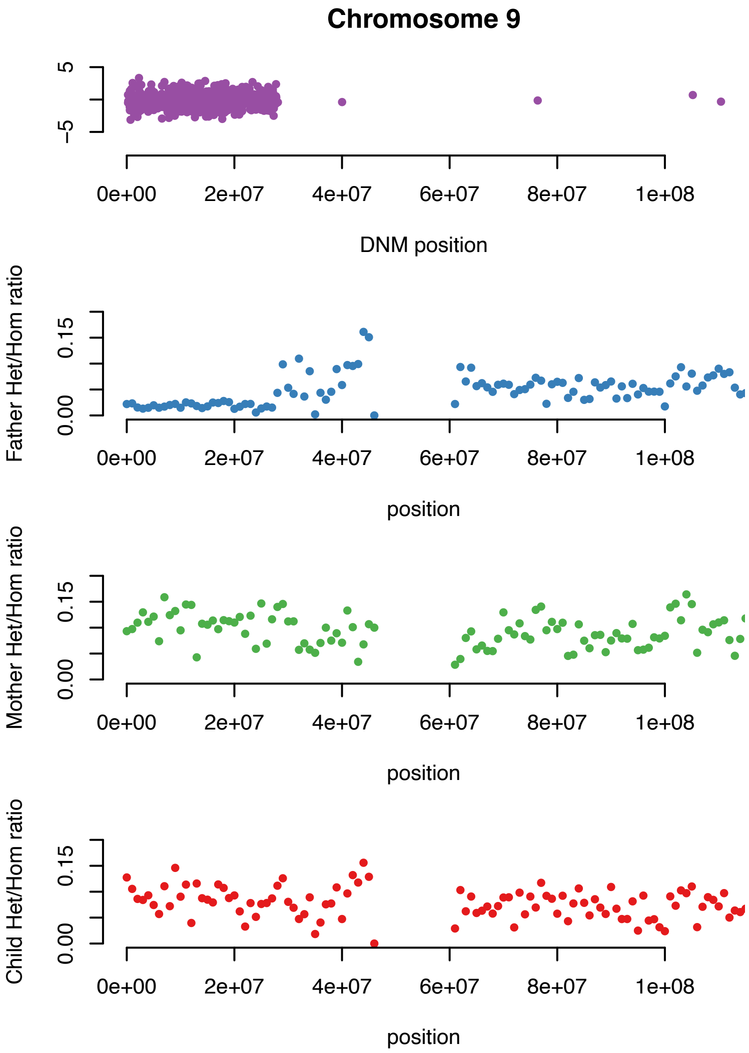

Supplemental Figure 7: Loss of transmitted allele example leading to false positive DNMs Top plot shows the location of the called DNMs in the child on chromosome 9. The plots below show the heterozygous/homozygous ratio in the Father, Mother and Child showing a loss of heterozygosity in the father in the same region the DNMs have been called.

Supplemental Tables

Supplemental Table 1: Trinucleotide mutation counts for 12 hypermutated individuals

Supplemental Table 3: Mutation probabilities for novel mutational signature SBSHYP

Supplemental Table 4: DNA repair genes with annotations taken from https://www.mdanderson.org/documents/Labs/Wood-Laboratory/human-dna-repair-genes.html (accessed January 2020)

ID	C>A	C>G	C>T	CpG>TpG	T>A	T>C	T>G
GEL_1	1.0E-158	7.9E-28	1.1E-28	1.9E-01	2.3E-30	5.9E-17	1.5E-19
GEL_2	4.2E-06	2.6E-68	3.7E-05	5.6E-02	5.9E-11	2.5E-64	4.9E-56
GEL_3	6.8E-01	1.4E-01	1.5E-01	8.8E-01	6.1E-01	3.6E-216	3.3E-01
DDD_1	2.2E-10	3.1E-51	2.1E-10	6.6E-01	9.1E-04	7.9E-16	1.8E-35
GEL_4	4.2E-06	1.0E-04	6.7E-11	2.0E-47	1.0E-08	3.4E-14	5.1E-04
GEL_5	4.6E-11	1.1E-08	2.1E-10	7.9E-02	5.1E-08	3.7E-05	1.6E-03
GEL_6	4.5E-09	1.9E-07	3.7E-05	4.7E-01	1.6E-12	6.5E-03	1.1E-05
GEL_7	6.8E-01	3.0E-01	3.7E-05	9.1E-31	4.5E-01	8.5E-02	1.0E+00
GEL_8	3.1E-07	8.9E-02	3.7E-05	3.1E-01	2.0E-05	4.9E-02	1.2E-02
GEL_9	1.9E-15	3.0E-01	7.7E-05	7.7E-01	8.1E-03	9.6E-03	8.3E-02
GEL_10	1.5E-04	1.9E-07	6.8E-02	5.6E-01	2.1E-02	7.1E-05	4.5E-02
GEL_11	9.3E-12	6.7E-01	6.4E-10	5.6E-01	1.3E-01	1.0E+00	2.6E-02

Supplemental Table 2: Corresponding p-values for enrichment of mutation type for each hypermutated individual. This is a two-sided Poisson test comparing the average number of mutations in each type across all individuals in the 100kGP cohort. These are demonstrated as colours in Figure 1b.

ID	Child disease	Genetic variant**	Parental chemotherapy exposure*
GEL_1	Epileptic encephalopathy	Father: 3:14165549 G>A homozygous<br>NM_004628.5(XPC):c.658C>T (p.Arg220Ter)<br>ClinVar ID: 550020<br>GnomAD allele frequency: 2.2e-5<br>Clinical diagnosis of xeroderma pigmentosum	NA
GEL_2	Multisystem developmental disorder	NA	Nephrotic syndrome: Cyclophosphamide, Chlorambucil (and immunosuppressants)
GEL_3	Intellectual disability	Father: 16:83139 G>A homozygous<br>NM_002434.4(MPG):c.403G>A (p.Ala135Thr)<br>ClinVar ID: absent<br>GnomAD allele frequency: 9.57e-5	NA
GEL_4	Multisystem developmental disorder, myelodysplasia	Child: 12:11885935 A>G mosaic heterozygous<br>NM_001987.5(ETV6):c.1162A>G (p.Asn388Asp)<br>ClinVar ID: absent<br>GnomAD allele frequency: 0 (absent)	NA
GEL_5	Pulmonary fibrosis	NA	Systemic lupus erythematosus: [Chemotherapy confirmed, drugs unknown]
GEL_6	Congenital myopathy	NA	NA
GEL_7	Intellectual disability	NA	NA

GEL_8	Abnormality of copper homeostasis	NA	Testicular cancer:<br>Drugs unknown
GEL_9	Intellectual disability	NA	Testicular cancer:<br>BEP (Bleomycin, etoposide and platinum)
GEL_10	Intellectual disability	NA	NA
GEL_11	Cataracts	NA	Cancer of long bones, intestinal tract, lung (secondary):<br>Drugs unknown
DDD_1	Global Developmental Delay, Microcephaly	NA	Hodgkins Lymphoma:<br>ABVD (Bleomycin-Dacarbazine-Doxorubicin-Vinblastine)<br>IVE (Iphosphamide, epirubicin and etoposide)

*prior to conception
** GRCh38 coordinates

Supplemental Table 5: Summary of putative mutagenic variants and parental pharmacological exposures for hypermutated individuals

MPG variant	alle frequency[1]	eA•T k_{rel}	Hx•T k_{rel}	Specificity eA/Hx	Reference
R120C[3]	5×10^{-4}	0.9	NR[2]	NR	Adhikari, Chetram et al. (2015)
Y127W	NR	0.13	NR	NR	O'Brien and Ellenberger (2003)
R141Q[3]	8×10^{-5}	0.8	NR	NR	Adhikari, Chetram et al. (2015)
Y159W	NR	0.37	NR	NR	O'Brien and Ellenberger (2003)
**A135T**	$\mathbf{1 \times 10^{-4}}$	**2.2**	**0.93**	**2.4**	**this work**
R138S	NR	1.1	0.78	1.3	Zhang and O'Brien (2015)
R141M	NR	1.0	1.0	1.0	Zhang and O'Brien (2015)
R145S	NR	1.0	0.32	3.1	Zhang and O'Brien (2015)
Y162W	NR	1.0	0.42	2.4	Hendershot and O'Brien (2017)
N169S[4]	NR	2.0	1.1	1.8	O'Brien and Ellenberger (2004)
R182M	NR	0.64	0.44	1.5	Zhang and O'Brien (2015)
R197S	NR	1.1	0.68	1.5	Zhang and O'Brien (2015)
K210M	NR	0.9	1.1	0.82	Zhang and O'Brien (2015)
K220M	NR	1.5	0.85	1.8	Zhang and O'Brien (2015)
K229M	NR	1.0	0.93	1.1	Zhang and O'Brien (2015)

Supplemental Table 6: Compilation of single turnover excision kinetics for MPG variants. Relative single-turnover glycosylase activity is reported as the ratio of the single turnover rate constant for the variant divided by that of the WT enzyme from the indicated reference. [1]Allele frequency from GnomAD. [2]NR, not reported. [3]R120C and R141Q are the most deleterious variants tested out of 8 rare alleles of MPG. R141Q and to a lesser extent R120C showed a modest increase in mutation frequency in a plasmid repair assay performed in HEK293 cells (Adhikari, Chetram et al. (2015)). [4]N169S shows a mutator phenotype when it is expressed in yeast (Eyler, Burnham et al. (2017), Connor, Wilson et al. (2005)).

ID	Number SNVs	Number Indels	SNV p-value	Paternal age	Maternal age	Paternal DNMs	Maternal DNMs	Phase p-value	Chemo code
MatCancer_1	86	5	0.014	(30,35]	(25,30]	14	8	0.095	Y
MatCancer_10	49	4	0.983	(35,40]	(30,35]	12	6	0.194	N
MatCancer_11	67	8	0.440	(30,35]	(30,35]	15	5	0.469	N
MatCancer_12	61	6	0.433	(25,30]	(30,35]	22	3	0.940	N
MatCancer_13	79	10	0.249	(35,40]	(30,35]	20	6	0.536	N
MatCancer_14	79	6	0.298	(35,40]	(35,40]	15	4	0.639	N
MatCancer_15	60	5	0.710	(30,35]	(30,35]	17	5	0.562	N
MatCancer_16	43	6	0.609	(20,25]	(20,25]	11	5	0.274	N
MatCancer_17	73	4	0.336	(35,40]	(30,35]	19	1	0.993	N
MatCancer_18	72	8	0.369	(35,40]	(30,35]	15	7	0.201	N
MatCancer_19	75	11	0.318	(35,40]	(35,40]	15	7	0.201	Y
MatCancer_2	66	4	0.464	(30,35]	(30,35]	24	3	0.958	N
MatCancer_20	72	10	0.324	(30,35]	(30,35]	18	5	0.605	N
MatCancer_21	64	6	0.779	(35,40]	(30,35]	16	2	0.934	N
MatCancer_22	94	6	0.155	(40,45]	(40,45]	20	6	0.536	Y
MatCancer_23	103	4	0.101	(45,50]	(35,40]	22	14	0.018	Y
MatCancer_24	68	6	0.553	(35,40]	(25,30]	17	4	0.720	N
MatCancer_25	43	3	0.745	(20,25]	(20,25]	11	3	0.633	Y
MatCancer_26	93	5	0.279	(45,50]	(30,35]	22	5	0.750	Y
MatCancer_27	88	5	0.176	(40,45]	(30,35]	17	6	0.407	N
MatCancer_3	62	3	0.892	(35,40]	(40,45]	16	3	0.829	Y
MatCancer_4	86	6	0.529	(45,50]	(35,40]	33	8	0.721	N
MatCancer_5	67	5	0.565	(35,40]	(25,30]	20	8	0.273	N
MatCancer_6	85	4	0.014	(25,30]	(30,35]	21	6	0.577	N
MatCancer_7	96	1	0.122	(40,45]	(40,45]	19	10	0.091	N
MatCancer_8	78	3	0.092	(30,35]	(20,25]	20	2	0.971	N
MatCancer_9	58	3	0.321	(25,30]	(20,25]	11	4	0.437	Y
PatCancer_1	49	5	0.966	(30,35]	(30,35]	12	5	0.842	N
PatCancer_10	94	8	0.005	(30,35]	(25,30]	24	3	0.118	N
PatCancer_11	45	10	0.713	(20,25]	(20,25]	11	3	0.619	N
PatCancer_12	99	10	0.143	(45,50]	(25,30]	21	7	0.727	N
PatCancer_13	42	6	0.955	(25,30]	(25,30]	9	6	0.968	N
PatCancer_14	55	7	0.800	(30,35]	(30,35]	15	3	0.407	Y
PatCancer_15	73	10	0.147	(30,35]	(20,25]	18	6	0.725	N
PatCancer_16	92	4	0.475	(55,60]	(25,30]	27	0	0.001	N
PatCancer_17	42	3	0.667	(20,25]	(15,20]	9	4	0.858	N
PatCancer_18	65	3	0.806	(40,45]	(30,35]	14	8	0.961	N
PatCancer_19	63	6	0.746	(35,40]	(30,35]	14	3	0.456	N
PatCancer_2	52	1	0.381	(20,25]	(15,20]	12	3	0.563	Y
PatCancer_20	54	4	0.333	(20,25]	(25,30]	14	7	0.925	N
PatCancer_21	54	4	0.828	(30,35]	(30,35]	12	2	0.367	N
PatCancer_22	80	8	0.287	(35,40]	(30,35]	16	8	0.934	Y
PatCancer_23	50	4	0.851	(25,30]	(30,35]	12	9	0.991	N
PatCancer_24	55	3	0.889	(35,40]	(30,35]	11	7	0.970	Y
PatCancer_25	51	4	0.952	(35,40]	(30,35]	7	5	0.968	N
PatCancer_3	99	6	0.129	(50,55]	(20,25]	26	6	0.411	N
PatCancer_4	73	5	0.025	(25,30]	(20,25]	20	6	0.646	N
PatCancer_5	64	4	0.195	(25,30]	(25,30]	9	3	0.732	N
PatCancer_6	70	5	0.721	(40,45]	(35,40]	7	5	0.968	N
PatCancer_7	48	5	0.426	(20,25]	(15,20]	17	4	0.484	N
PatCancer_8	55	5	0.624	(25,30]	(30,35]	10	6	0.954	N
PatCancer_9	100	5	0.001	(30,35]	(30,35]	29	8	0.556	N

Supplemental Table 7: Individuals with a parent with a cancer diagnosis reported in hospital episode statistics prior to conception. Prefix of ID indicates whether the mother (MatCancer) or father (PatCancer) is the parent with cancer diagnosis. Number of SNVs and Indels refers to the DNM count in the child. The SNV p-value is test for if the number of SNVs is significantly greater than expected given parental age. Paternal and maternal age are given in 5 year bins. The number of paternal and maternal DNMs are the count of DNMs that phased paternally and maternally. The phase p-value is testing if proportion of DNMs that phase paternally is different to overall proportion across 100kGP dataset. Chemo code indicates whether the parent also has an ICD10 code for chemotherapy yes (Y) or no (N).

Variant Subset	Csq	Genotype	Paternal count	Paternal Effect	Paternal p-value	Maternal count	Maternal effect	Maternal p-value
All DNA repair	nonsyn	het	5857	0.12	0.65	5903	-0.08	0.79
	PTV	het	1203	0.28	0.36	1150	-0.12	0.70
	nonsyn	hom	78	1.50	0.19	71	0.59	0.61
	PTV	hom	13	-1.31	0.64	11	1.52	0.62
Subset DNA repair	nonsyn	het	3075	0.07	0.77	2918	0.12	0.62
	PTV	het	432	0.03	0.95	388	0.44	0.39
Germline cancer	nonsyn	het	103	1.28	0.19	97	-0.54	0.60
	PTV	het	41	1.27	0.41	35	-1.87	0.26

Supplemental Table 8: Impact of parental rare variants in DNA repair genes on germline mutation rate. Effect estimates and corresponding p-values from 8 regression models on three subsets of variant groups. Csq: consequence of variants examined where ‘nonsyn’ refers to nonsynonymous variants and PTV refers to a subset of these of just protein truncating variants. Genotype details whether the variants considered were ‘het’: heterozygous or ‘hom’: homozygous. Paternal count refers to the number of variants found in this subset in paternal genomes and maternal count refers to the equivalent for mothers.

MAF bin	LD group	Maternal h^2	Maternal SE	Paternal h^2	Paternal SE
0.001-0.01	low	0.151	0.195	0.337	0.167
	High	-0.008	0.205	0.181	0.136
0.01-0.05	low	-0.018	0.083	10^{-6}	0.070
	high	0.026	0.032	10^{-6}	0.026
0.05-1	low	-0.074	0.061	10^{-6}	0.051
	high	-0.002	0.032	0.008	0.027
TOTAL	-	0.071<br>p = 0.21	0.255	0.526<br>p = 0.095	0.165
Number of individuals	-	6329		6352	

Supplemental Table 9: Maternal and paternal SNP heritability of residuals of number of dnSNVs after correcting for parental age, hypermutation status and data quality. Results from GREML-LDMS binned on three minor allele frequency (MAF) bins and two LD groups. High LD refers to variants with LD > median LD and low LD refers to variants with LD < medianLD. Maternal heritability has negative estimates as this was run without being constrained to positive numbers due to estimates not converging otherwise. SE refers to standard error of the h^2 estimate. Performed on a subset of individuals with white british ancestry.

Supplementary References

Adhikari, S., M. A. Chetram, J. Woodrick, P. S. Mitra, P. V. Manthena, P. Khatkar, S. Dakshanamurthy, M. Dixon, S. K. Karmahapatra, N. K. Nuthalapati, S. Gupta, G. Narasimhan, R. Mazumder, C. A. Loffredo, A. Uren and R. Roy (2015). "Germ line variants of human N-methylpurine DNA glycosylase show impaired DNA repair activity and facilitate 1,N6-ethenoadenine-induced mutations." J Biol Chem **290**(8): 4966-4980.

Connor, E. E., J. J. Wilson and M. D. Wyatt (2005). "Effects of substrate specificity on initiating the base excision repair of N-methylpurines by variant human 3-methyladenine DNA glycosylases." Chem Res Toxicol **18**(1): 87-94.

Eyler, D. E., K. A. Burnham, T. E. Wilson and P. J. O'Brien (2017). "Mechanisms of glycosylase induced genomic instability." PLoS One **12**(3): e0174041.

Hendershot, J. M. and P. J. O'Brien (2017). "Search for DNA damage by human alkyladenine DNA glycosylase involves early intercalation by an aromatic residue." J Biol Chem **292**(39): 16070-16080.

O'Brien, P. J. and T. Ellenberger (2003). "Human alkyladenine DNA glycosylase uses acid-base catalysis for selective excision of damaged purines." Biochemistry **42**(42): 12418-12429.

O'Brien, P. J. and T. Ellenberger (2004). "Dissecting the broad substrate specificity of human 3-methyladenine-DNA glycosylase." J Biol Chem **279**(11): 9750-9757.

Zhang, Y. and P. J. O'Brien (2015). "Repair of Alkylation Damage in Eukaryotic Chromatin Depends on Searching Ability of Alkyladenine DNA Glycosylase." ACS Chem Biol **10**(11): 2606-2615.
