# Supplementary material for: Genetic and chemotherapeutic influences on germline hypermutation
Source: Nature. 2022 May 11;605(7910):503–8. doi: 10.1038/s41586-022-04712-2 (PMC9117138; doi:10.1038/s41586-022-04712-2)
Supplement: Supplementary file 2 — Reporting Summary [file 41586_2022_4712_MOESM2_ESM.pdf]

nature research

Corresponding author(s): Matthew Hurles

Last updated by author(s): Jun 14, 2021

Reporting Summary

Nature Research wishes to improve the reproducibility of the work that we publish. This form provides structure for consistency and transparency in reporting. For further information on Nature Research policies, see Authors & Referees and the Editorial Policy Checklist.

Statistics

For all statistical analyses, confirm that the following items are present in the figure legend, table legend, main text, or Methods section.

n/a	Confirmed	
☐	☒	The exact sample size (*n*) for each experimental group/condition, given as a discrete number and unit of measurement
☐	☒	A statement on whether measurements were taken from distinct samples or whether the same sample was measured repeatedly
☐	☒	The statistical test(s) used AND whether they are one- or two-sided<br>*Only common tests should be described solely by name; describe more complex techniques in the Methods section.*
☐	☒	A description of all covariates tested
☐	☒	A description of any assumptions or corrections, such as tests of normality and adjustment for multiple comparisons
☐	☒	A full description of the statistical parameters including central tendency (e.g. means) or other basic estimates (e.g. regression coefficient) AND variation (e.g. standard deviation) or associated estimates of uncertainty (e.g. confidence intervals)
☐	☒	For null hypothesis testing, the test statistic (e.g. *F*, *t*, *r*) with confidence intervals, effect sizes, degrees of freedom and *P* value noted<br>*Give P values as exact values whenever suitable.*
☒	☐	For Bayesian analysis, information on the choice of priors and Markov chain Monte Carlo settings
☒	☐	For hierarchical and complex designs, identification of the appropriate level for tests and full reporting of outcomes
☐	☒	Estimates of effect sizes (e.g. Cohen's *d*, Pearson's *r*), indicating how they were calculated

Our web collection on statistics for biologists contains articles on many of the points above.

Software and code

Policy information about availability of computer code

Data collection	NA
Data analysis	Analyses were performed primarily in R (4.0.1). Phasing of mutations was performed with a custom Python (3) script available at https://github.com/queenjobo/PhaseMyDeNovo. Signature extraction was performed using SigProfiler (v1.0.17). Details of software used for sequence alignment, variant calling and de novo mutation calling are given in the Methods. Analysis of gels was done with ImageQuant TL 7.0 (Cytiva).

For manuscripts utilizing custom algorithms or software that are central to the research but not yet described in published literature, software must be made available to editors/reviewers. We strongly encourage code deposition in a community repository (e.g. GitHub). See the Nature Research guidelines for submitting code & software for further information.

Data

Policy information about availability of data

All manuscripts must include a data availability statement. This statement should provide the following information, where applicable:

- Accession codes, unique identifiers, or web links for publicly available datasets
- A list of figures that have associated raw data
- A description of any restrictions on data availability

Sequence and variant-level data and phenotypic data for the DDD study data are available from the European Genome-phenome Archive (EGA; https://www.ebi.ac.uk/ega/) with study ID EGAS00001000775. This is under managed access to ensure that the work proposed by the researchers is allowed under the study's ethical approval.
Sequence and variant-level data (including the de novo mutations dataset) and phenotypic data from the 100,000 Genomes Project can be accessed by application to Genomics England Ltd following the procedure outlined at: https://www.genomicsengland.co.uk/about-gecip/joining-researchcommunity/
Genome Aggregation Database (gnomAD v2.1.1; https://gnomad.broadinstitute.org/)

Catalogue of Somatic Mutations in Cancer (v3.1; https://cancer.sanger.ac.uk/)
ClinVar (https://www.ncbi.nlm.nih.gov/clinvar/)

Field-specific reporting

Please select the one below that is the best fit for your research. If you are not sure, read the appropriate sections before making your selection.

☒ Life sciences ☐ Behavioural & social sciences ☐ Ecological, evolutionary & environmental sciences

For a reference copy of the document with all sections, see nature.com/documents/nr-reporting-summary-flat.pdf

Life sciences study design

All studies must disclose on these points even when the disclosure is negative.

Sample size	This is an observational study: the sample size is 7,930 exome sequenced parent-child trios from the DDD study and 13,949 whole genome sequenced parent-child trios from the 100,000 Genome Project. No power calculations were done prior to analyses; we used all available samples.
Data exclusions	We excluded 12 trios in the 100,000 Genomes Project with a high false positive rate of de novo mutations as outlined in the Methods.
Replication	There was no replication in this study as it was focused on specific outliers in an observational study which is unable to be replicated. However these outliers were identified in two separate studies. Analyses performed across the whole cohort were not replicated although results were compared and found to be very similar to previous published results from other studies.
Randomization	There was no randomization in this study as it was not applicable since we were identifying specific outliers in an observational study.
Blinding	There was no blinding in this study as it was not applicable as it was an observational study focussed on genetic data.

Reporting for specific materials, systems and methods

We require information from authors about some types of materials, experimental systems and methods used in many studies. Here, indicate whether each material, system or method listed is relevant to your study. If you are not sure if a list item applies to your research, read the appropriate section before selecting a response.

Materials & experimental systems

n/a	Involved in the study	
☒	☐	Antibodies
☒	☐	Eukaryotic cell lines
☒	☐	Palaeontology
☒	☐	Animals and other organisms
☐	☒	Human research participants
☒	☐	Clinical data

Methods

n/a	Involved in the study	
☒	☐	ChIP-seq
☒	☐	Flow cytometry
☒	☐	MRI-based neuroimaging

Human research participants

Policy information about studies involving human research participants

Population characteristics	The Deciphering Developmental Disorders study consists of individuals with severe developmental disorders recruited along with their parents. The rare disease arm of the 100,000 Genomes Projects consists of individuals with rare disease (that fall into 15 rare disease domains) recruited along with their parents (see https://www.nature.com/articles/s41586-020-2434-2#Sec21 for details)
Recruitment	Recruitment differed for the two cohorts:<br><br>Deciphering Developmental Disorders (DDD): Patients with severe, undiagnosed developmental disorders were recruited from 24 regional genetics services within the United Kingdom National Health Service and the Republic of Ireland. These analyses involve 7,930 trios who have been analyzed in previous publications. Patients typically had some prior genetic testing (e.g. an array or a single gene test) before recruitment into the study.<br><br>100,00 Genomes Project: Study participants were enrolled by one of three mechanisms between December 2012 and March 2017 under the overall coordination of the National Institute for Health Research BioResource (NBR) at Cambridge University Hospitals. Patients with rare diseases and their close relatives were enrolled into 15 rare disease domains approved by the Sequencing and Informatics Committee of the NBR. Participants in the rare disease domains were recruited mainly at NHS Hospitals in the United Kingdom, but also at hospitals overseas.

Ethics oversight	DDD: The study was approved by the UK Research Ethics Committee (10/H0305/83 granted by the Cambridge South Research Ethics Committee, and GEN/284/12 granted by the Republic of Ireland Research Ethics Committee).<br><br>100,000 Genomes Project: All participants provided written informed consent, either under the East of England Cambridge South national research ethics committee (REC) reference no. 13/EE/0325 or under ethics for other REC-approved studies.

Note that full information on the approval of the study protocol must also be provided in the manuscript.
